# Enterovirus D68-associated respiratory and neurological illness in Spain, 2014–2018

**DOI:** 10.1080/22221751.2019.1668243

**Published:** 2019-10-01

**Authors:** Rubén González-Sanz, Irene Taravillo, Jordi Reina, Ana Navascués, Antonio Moreno-Docón, Maitane Aranzamendi, María Pilar Romero, Margarita del Cuerpo, Carmen Pérez-González, Sonia Pérez-Castro, Almudena Otero, María Cabrerizo

**Affiliations:** a Enterovirus Unit, National Centre for Microbiology, Instituto de Salud Carlos III, Madrid, Spain; b Hospital Son Espases, Palma de Mallorca, Spain; c Complejo Hospitalario de Navarra, Navarra, Spain; d Hospital Virgen de la Arrixaca, Murcia, Spain; e Hospital Universitario Cruces, Biocruces Bizkaia Health Research Institute, Bilbao, Spain; f Hospital La Paz, Madrid, Spain; g Hospital Santa Creu i Sant Pau, Barcelona, Spain; h Hospital Dr. Negrín, Las Palmas de Gran Canaria, Spain; i Servicio de Microbiología, Complexo Hospitalario Universitario de Vigo (CHUVI), Sergas, Vigo, Spain

**Keywords:** Enterovirus, enterovirus D68, respiratory infections, acute flaccid paralysis, phylogenetic analysis

## Abstract

During 2014, enterovirus D68 (EV-D68) outbreaks were described globally, causing severe respiratory diseases in children and, in some cases, subsequent paralysis. In this study, the type characterization of enterovirus (EV) detected in respiratory illnesses and the epidemiology and clinical association of EV-D68 infections in Spain over a five-year period were described. A total of 546 EV-positive samples from hospitalized patients with respiratory infections were included. EV-D68 was the most frequently detected type (46.6%, 191/410 typed EV). Other EV from species A (25.1%), B (27.8%) and C (0.5%) were also identified. EV-D68 infections were more associated with bronchitis while EV-A/B types were more frequent in upper respiratory illness (*p* < 0.01). EV-D68 was also detected in patients with neurological symptoms (nine meningitis/meningoencephalitis and eight acute flaccid paralysis cases). Phylogenetic analysis of 3′-VP1 region showed most Spanish EV-D68 sequences from 2014 to 2016 belonged to subclades B2/B3, as other American and European strains circulating during the same period. However, those detected in 2017 and 2018 clustered to the emerged subclade D1. In summary, different EV can cause respiratory infections but EV-D68 was the most prevalent, with several strains circulating in Spain at least since 2014. Association between EV-D68 infection and neurological disease was also described.

## Introduction

Enteroviruses (EV) are responsible for a wide range of diseases in humans including febrile illness, myocarditis and neurologic illness, such as aseptic meningitis, encephalitis or paralysis [1]. EV can be also found in the respiratory tract and some types, especially from species C and D cause upper and lower respiratory symptoms [2]. Since the first isolation in 1962 [3], Enterovirus D68 (EV-D68) has been associated almost exclusively with respiratory diseases. It shares similar biological characteristics with rhinoviruses, which are currently classified together with EV in the genus *Enterovirus* within the *Picornaviridae* family [4]. EV-D68 has acid sensitivity and grows at lower optimal temperatures in some cell lines than other EV [5]. Before 2014, only a few outbreaks or sporadic cases of EV-D68 infections had been reported in different parts of the world [6–8]. However, in the late summer of that year, a large-scale outbreak of severe respiratory illness associated with EV-D68 was noted in the United States (US) and Canada [9,10]. Moreover, there was a significant increase of cases with neurological complications after the respiratory episode, mainly acute flaccid myelitis [11].

Since 2014, surveillance of EV-D68 infections is recommended and several studies have been published in Europe and other parts of the world regarding EV-D68 circulation, some reflecting an increase in detection in recent years [12–16]. Additionally, cases of paralysis or severe myelitis in children, in which EV-D68 was the only etiologic agent detected, were reported in different countries, including Spain [17–21]. Regarding respiratory diseases, in our country most of detected EV were not usually characterized, and the first reports of EV-D68 detection were published in 2015 [22–24]. Therefore, the objectives of the present study were first, to identify the genotype of the EV detected in clinical samples collected from hospitalized patients with different respiratory diseases over a 5-year period, and second, to describe the molecular epidemiology and clinical characteristics of EV-D68 infections in Spain.

## Patients and methods

*Clinical samples*. Between April 2014 and December 2018, the Enterovirus Reference Laboratory of the Spanish National Centre for Microbiology (CNM) received a total of 546 EV-positive samples from patients with different respiratory diseases for type characterization. Patients had been admitted in 17 hospitals from 11 Spanish provinces (Vizcaya, Madrid, Murcia, Balearic Islands, Zaragoza, Pontevedra, Barcelona, Navarra, Canary Islands, Cáceres and Alicante). Clinical data about diagnosis, underlying diseases, intensive care unit (ICU) stay and coinfections with virus other than EVs were kindly provided by the hospitals included in this study. The institutional ethics board approved the study and informed consent was obtained from parents or tutors in those cases needed.

Specimens were 526 throat/nasopharyngeal swabs, 18 bronchoalveolar lavages, and 2 stools. The number of EV-positive respiratory samples received each year during the study varied: 30 in 2014, 106 in 2015, 258 in 2016, 57 in 2017, and 95 in 2018.

*Enterovirus genotyping*. RNA was extracted from clinical samples using the QIAamp Viral RNA Mini Kit (QIAGEN, Germany**).** Genotyping was performed using a previously described RT-nested PCRs in 3’-VP1 region for EV species A, B and C [25] and subsequent sequencing. In addition, a specific RT-nested PCR for EV-D68 detection in the same region 3`-VP1 was designed and evaluated. Primers used in this case were: outer sense, 5’-CATACCTTAGRTTTGATGCTG and outer antisense, 5’-CCATTGAATYCCTGGRCCTTC; inner sense, 5’- GTACCMACTGGTGCTCTTAC and inner antisense, 5’- CTGATTGCCARTCCACATAG. PCR reaction mix, cycling programme, and sequencing conditions were the same as previously described for species A, B and C RT–PCR [25]. An EV-D68 isolate cultured in RD cells was used to validate the designed RT–PCR. The virus titre was determined by the median cell culture infective dose (CCID_50_/ml) and calculated by the Kärber method [26]. To determine the lower limit of detection (LoD) of the assay, serial 10-fold dilutions of the virus, containing 10^7^ CCID_50_/ml, were analysed. A set of 15 EV strains from species A to C and four EV-D68 isolates (obtained in 2010–2011 by inoculating RD cells with respiratory samples from hospitalized patients with respiratory illnesses) was analysed for testing the RT-PCR efficiency (Table 1).

**Table 1.** Panel of enterovirus (EV), parechovirus (HPeV) and rhinoviruses (RV) types used for validation of the EV-D68 RT-nested PCR assay.

Virus	EV-D68 RT-nested PCR
EV-A: CV-A6, CV-A16, CV-A10, EV-A71	negative
EV-B: E-5, E-6, E-9, E-11, E-13, E-20, E-30, CV-B3, CV-B4	negative
EV-C: CV-A24, PV1SL	negative
EV-D68 isolates:	
SP_AGO10_50323_R	positive
SP_SEP10_50324_R	positive
SP_JUN11_50346_R	positive
SP_OCT10_50439_R	positive
RV-A, RV-C	negative
HPeV-1, HPeV-3	negative

*Phylogenetic analysis*. A total of 201 EV-D68 sequences obtained in this study and detected between 2014 and 2018 were included in a phylogenetic analysis with different EV-D68 sequences available in GenBank in the same 3’-VP1 region from American, Asian and European countries (*N* = 89). The prototype strain FermonUS/1962 was included as the outgroup. The phylogenetic tree was constructed with MEGA 7.0 software using a neighbour-joining distance method under the maximum composite likelihood substitution model with 1,000 bootstrap resamplings. The sequences obtained in this study have been deposited in GenBank under accession numbers MN403103 - MN403299.

*Statistical analysis*. Clinical characteristics and laboratory variables were compared using the Student’s t test and Fisher exact test. A 2-sided value of *p* < 0.05 was considered statistically significant.

## Results

*LoD and validation of the designed EV-D68 RT-nested PCR*. Using serial dilutions of the assay EV-D68-positive control, the minimal amount of virus detected by the specific PCR assay was 1 CCID_50_/ml. A set of 15 EV strains from species A to C and four EV-D68 isolates was analysed for testing the RT–PCR efficiency. Only EV-D68-positive samples were amplified (Table 1). The cross-reactivity with other viruses was also studied. Rhinoviruses (RV) or human parechoviruses (HPeV) were not detected.

**Figure 1.**
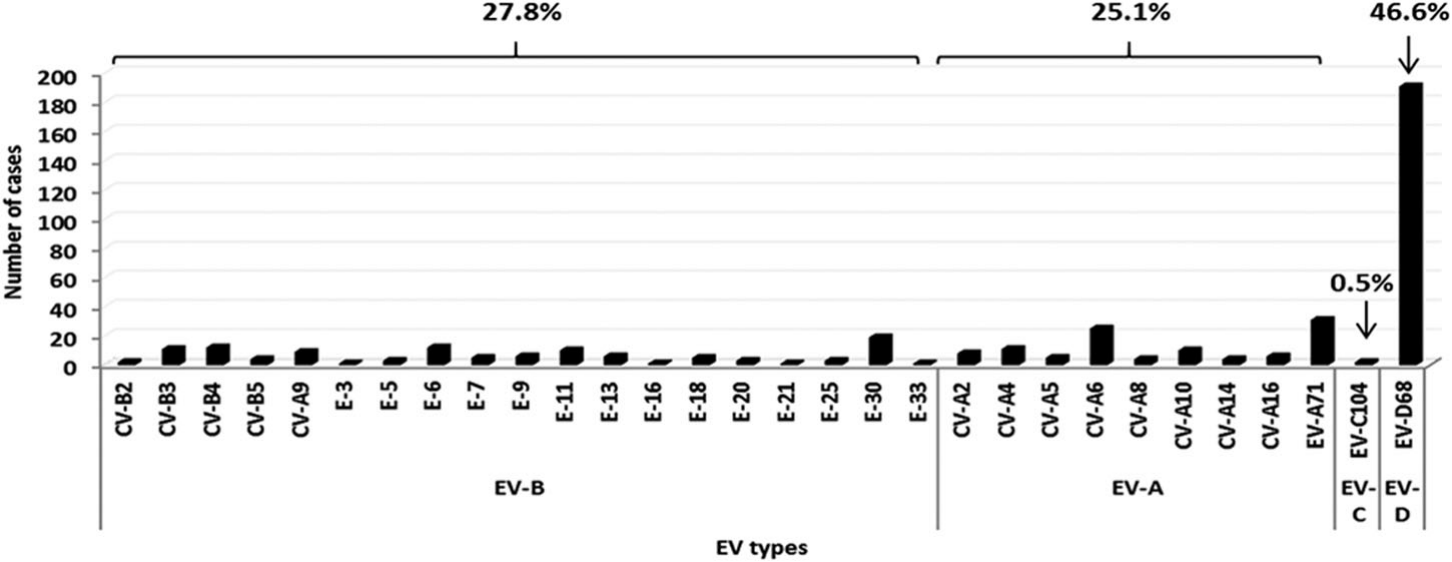
Enterovirus genotypes detected in the respiratory specimens included in this study.

*EV genotype identification*. From the total of 546 EV-positive respiratory samples included in the study, 410 (75.1%) were successfully amplified with EV-A, B, C or D68 RT-nested PCRs. RV was identified in 41 samples and in the remaining 95 untyped EV, the presence of EV-D68 was excluded by using a RT-nested PCR in 5´-non-coding region of the EV genome [27] and subsequent sequencing. EV-D68 was the most frequent serotype detected (*N* = 191, 46.6%), followed by EV-A71 (*N* = 31, 7.6%), coxackievirus (CV)–A6 (*N* = 25, 6.1%), echovirus (E)-30 (*N* = 19, 4.6%) and E-6 (*N* = 12, 2.9%). Other EV types were also identified in minor proportion (Figure 1). Overall, the distribution of EV species was 46.6% for EV-D, 27.8% for EV-B, 25.1% for EV-A and 0.5% for EV-C (Figure 1).

**Figure 2.**
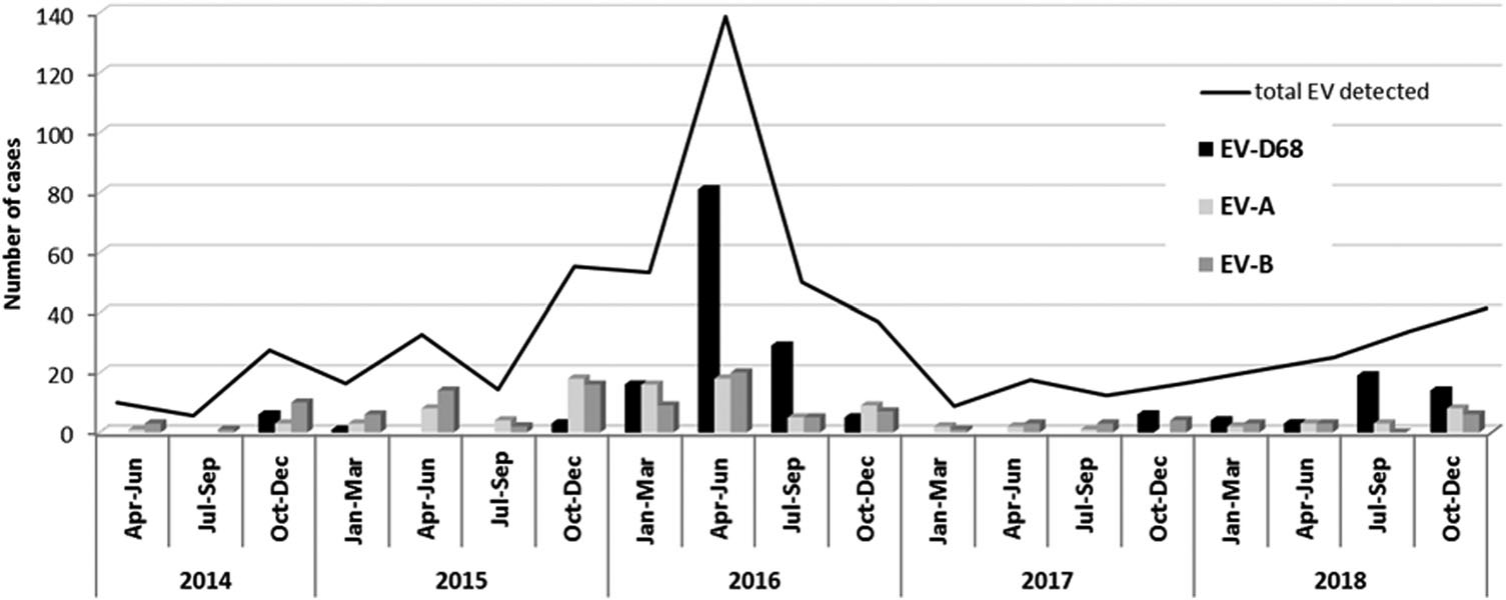
Seasonal distribution of EV-A, EV-B and EV-D68 infections in respiratory syndromes over the 5-year study period.

*Epidemiological and clinical characteristics of EV-D68 infections: comparison with EV-A or B infections*. Overall, EV-A and B types were detected throughout the year whereas EV-D68 was identified during the cold months (from October to March) ranged from 20 to 54% of the total EVs in those periods. In 2016 and 2018, in addition, a high incidence of EV-D68 infections was detected between April and September (warm months) with 121 and 22 cases which represented 74.2 and 70.9% respectively of the total EV typed in those months (Figure 2).

In 175/191 (91.6%) EV-D68 infections, the virus was the only known respiratory infectious agent detected in the samples. RV, human adenovirus (HAdV) and bocavirus (HBoV) were co-detected in nine, three and two of the EV-D68 patients, respectively. In addition, one patient was co-infected together with HAdV and HBoV, and another with RV, HAdV and human parainfluenza virus (HPIV). However, coinfection with one (*N* = 30), two (*N* = 11), three (*N* = 1) or even four (*N* = 2) respiratory viruses was identified in 44 out of 217 EV-A or B-positive patients (20.3% vs. 8.4% in EV-D68 infections, *p* = 0.000001), RV being the most frequent (*N* = 20). Also identified were respiratory syncytial virus, human metapneumovirus, HAdV, HBoV, HPIV, human coronavirus OC43 and influenza A virus.

Clinical data were available in 346 characterized EV infections: 172 were EV-D68-positive patients, 138 children (mean age, 3.2 ± SD 3.1 years, interquartile range 3.3) and 34 adults (mean age 57.2 ± SD 19.1 years, interquartile range 29.8); 174 were patients with EV-A/B infections, 164 children (mean age 2.1± SD 2.1 years, interquartile range 2.1) and 10 adults (mean age 65.4 ± SD 16.6 years, interquartile range 25.9). Bronchitis were more frequently diagnosed in EV-D68-infected children compared to EV-A/EV-B-positive patients (*p* < 0.01), whereas EV-A or EV-B genotypes were mainly detected in upper respiratory tract infections (*p* < 0.01) (Table 2). There were no significant differences in the proportion of patients who were admitted to the ICU when comparing EV-D68 with EV-A or B-positive patients (23/172, 13.4% vs 18/174 10.3%, *p* = 0.3904). All patients but two admitted to ICU were children.

**Table 2.** Comparison of the clinical symptoms between EV-D68- and EV-A or EV-B.

Respiratory symptoms	EV-D68-infected patients (*N *= 172)	EV-A or B-infected patients (*N *= 174)	*p* value
Bronchospasm/asthma	17 (9.9%)	10 (5.7%)	0.1587
Bronchiolitis	17 (9.9%)	17 (9.8%)	0.9720
Bronchitis	38 (22.1%)	11 (6.3%)	0.0000
Pneumonia	36 (20.9%)	35 (20.1%)	0.8524
Acute respiratory distress	38 (22.1%)	27 (15.5%)	0.1205
Upper respiratory tract infections	20 (11.6%)	62 (35.6%)	0.0000
Pharyngitis/tonsillitis	6 (3.5%)	12 (6.9%)	0.1638

Regarding previous clinical history, 49 out of 346 patients had an underlying disease (leukemia, multiple myeloma, lymphoma, cystic fibrosis, liver transplantation, congenital heart disease, and asthma or recurrent wheezing). Of them, 28/172 patients were infected by EV-D68 (16.3%) and 21/174 by EV-A or B (12.1%) (*p* = 0.1654). Only 4 of these patients required admission to the ICU.

## EV-D68 infection in non-respiratory diseases

During the routine EV genotyping in non-respiratory diseases (neurological, mucocutaneous and systemic diseases), EV-D68 was identified in 11 patients with febrile syndrome and 17 with specific neurological symptoms. All patients except two were children between one month and 13 years, and EV-D68 was the only pathogen detected. Regarding patients with neurological symptoms, eight children and one adult were diagnosed with meningitis or meningoencephalitis and the remaining eight children were finally described as acute flaccid paralysis (AFP) cases. In all but two patients, EV-D68 was detected in respiratory or/and stool samples. However, EV-D68 could be detected in CSF samples from those two patients (one with aseptic meningitis and one with meningoencephalitis). AFP cases were notified through the national AFP Surveillance System, all required admission to the ICU and all but two presented respiratory symptoms previously. One child with severe meningoencephalitis was also in ICU. EV-D68 AFP cases were identified throughout the study period (one case in 2015, four cases in 2016, one case in 2017 and two cases in 2018).

**Figure 3.**
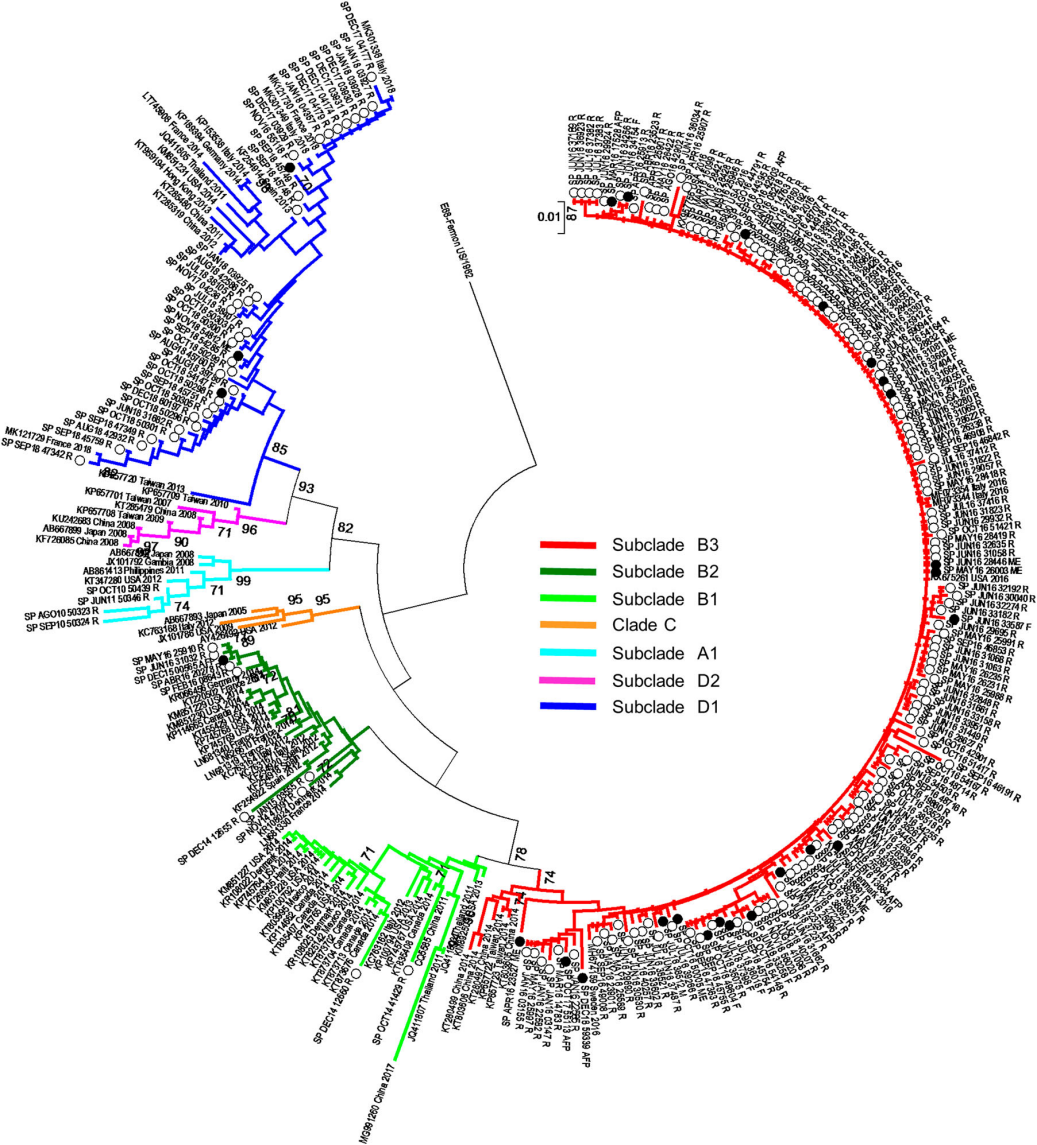
Phylogenetic analysis of 290 EV-D68 sequences in 3´-VP1 region (445 bp) from Spain, and representatives of different clades worldwide. MEGA 7.0 software was used to construct the neighbour joining and maximum composite likelihood tree. Only bootstra*p* values > 70% are shown. EV-D68 prototype strain Fermon was used as the outgroup. Isolates are indicated by the GenBank accession number, country and year of isolation. Spanish strains from 2014 to 2018 are represented by circles and indicated by country abbreviation, month and year of isolation and clinical manifestation. White circles represent respiratory-associated sequences and black circles represent neurological-associated sequences. Abbreviations for clinical manifestations are as follows: R-Respiratory disease, M-Meningitis, ME-Meningoencephalitis AFP-Acute flaccid paralysis, F-Fever.

*EV-D68 phylogenetic analysis*. EV-D68 phylogenetic tree in VP1 region (Figure 3) showed that almost all Spanish sequences from 2014 to 2016 belonged to the clade B, according to the classification previously described [7], and were genetically related to the majority of the strains detected in the US and Canada outbreaks during 2014 as well as sequences isolated in other European, American and Asian countries during 2009–2016. Spanish sequences from 2014 and 2015 clustered in subclade B2 whereas those detected in 2016 belonged to the subclade B3. However, most of the EV-D68 sequences from 2017 and 2018 (35/51, 68.6%) clustered in the recently described subclade D1 [28–30]. Finally, strains collected in our laboratory before 2014 (2010–2011) fell into clade A (Figure 3). Regarding to clinical manifestations, most neurological cases were B3 strains although B2 or D1 were also detected in two patients with these symptoms.

## Discussion

Since 2014, an increasing number of EV-D68 outbreaks associated with severe respiratory diseases, mainly in children, have been occurring in different countries, first in the US and Canada, followed by Europe and Asia. Concurrently, clusters of cases with neurological complications such as myelitis or acute flaccid paralysis were reported in the same geographical areas [9–21].

In Spain, EV surveillance in respiratory infections was not used to be routinely carried out. During 2014, however, the Enterovirus Laboratory of CNM started receiving EV-positive respiratory samples for genotyping. Our laboratory had already developed tools for genotyping EV species A, B and C but not for species D, in which serotype D68 belongs. Therefore, a specific RT–PCR for EV-D68 was designed. Analysis of EV-positive specimens collected from April 2014 to December 2018 from Spanish hospitalized patients with respiratory illnesses confirmed the presence of EV-D68 in almost half of the total characterized EV. Other EV types from species A, B and C were also detected at different rates. EV-A and B are occasionally detected in respiratory samples and their involvement in the respiratory pathology is not as clear as in the case of EV-D68 and EV-C104, EV-C105 or EV-C109 [2,5–16,31,32]. This is in agreement with the fact that in our study, in most cases, EV-D68 was the single infectious agent. In EV-A and B infections, however, the number of co-infections with other known respiratory virus was significantly higher which could mean that in those cases, EV could be an incidental finding. Furthermore, most of the identified EV-A and B types were those predominantly circulating in Spain during the same period of time (data not published). However, as there are few reports about which EV-A or B types are presented in respiratory symptoms, further studies are necessary to confirm or not their involvement in those pathologies. Although hospitals did not refer any bacterial infection in these patients, a possible limitation of our study could be the lack of common criteria from all of them testing the samples searching for different viruses or bacteria. Therefore, some coinfection positive cases could be missing.

Regarding seasonal distribution, EV-D68 cases were identified sporadically in cold months during the four years of study. In 2016 and 2018, however, it circulated almost throughout the year, with a higher incidence in spring and summer. This seasonal distribution, in which EV-D68 has periods of low circulation followed by others with high incidence, has been reported in other countries [29,30,33–35], and suggest epidemics of EV-D68 in 2016 and 2018, which was confirmed by phylogenetic analyses of partial VP1 sequences showing increased genetic diversity. The phylogenetic tree showed that most of the Spanish EV-D68 sequences detected during 2016 clustered to the recently described lineage B3 [33], while strains from 2014 and 2015 belonged to subclade B2. Similar results have been obtained with other studies [34,35], suggesting that all the 2016 outbreaks would be originated by the emergence of the clade B3 becoming predominant and replacing previously circulating clade A and subclades B1/B2. Another hypothesis might be that EV-D68 from clade B1/B2 caused non-symptomatic infections whose detection would have been underestimated since all included samples in this study were from hospitalized patients. Furthermore, most of the EV-D68 detected between November 2017 and December 2018 belonged to a new emerged subclade D1 detected firstly in China in 2016 and in Italy and France during 2018 [28–30]. These findings indicate that although EV-D68 epidemics can be due to the emergence of new lineages, multiple clades have been circulating simultaneously worldwide from 2010 to the present day.

As previously reported [16], most of the EV-D68 infections were detected in young children. However, there were also adult patients infected, half of them older than 65. Interestingly, 66% of sequences from 2017–2018 which belonged to subclade D1 were identified in adult patients with respiratory symptoms, suggesting a possible different epidemiology of this new strain. These findings had already been observed in previous studies [30].

Clinical data were available in most of the cases included in this study. In other studies [16] EV-D68 was detected in patients diagnosed with different respiratory diseases, ranging from a common cold with fever to basal pneumonia, but in our series, EV-D68 infection seemed to be more associated with bronchitis than with other respiratory presentations. By contrast, EV from species A or B were more frequently detected in upper respiratory tract infections (*p* < 0.01). Also, there was no significant difference in the proportion of patients from each group admitted to the ICU in contrast to those reported by other authors who observed a higher incidence of severe disease among patients infected by EV-D68 [33,35].

In addition to respiratory infections, EV-D68 has been associated with neurological complications in recent years [11,17–21]. During our study-period, this virus was identified in 16 children and one adult with neurological symptoms. Of them, eight were diagnosed as AFP cases. The association between EV-D68 and AFP or AFM is now accepted, although causality has not yet been proven. Regardless, the recent publication of a mouse model, in which mice inoculated with EV-D68 developed symptoms of myelitis, as well as several reports evaluating the evidence for a causal relationship supported this association [36–39]. Indeed, the World Health Organization and the Pan American Health Organization have recently recommended testing EV-D68 on respiratory samples in cases of AFP/AFM, both for case management and for surveillance purposes [40]. Furthermore, in the present study, EV-D68 was detected in CSF specimens from two patients with signs of meningoencephalitis, reinforcing the neurotropism of this EV. Finally, all neurological EV-D68 strains belonged to subclades B2/B3 or D1. These emerging genotypes are considered to have more neurotropic potential than A or C strains [30,36,37].

In conclusion, EV-D68 infections have been confirmed as a cause of mild lower respiratory illnesses in Spain, mainly in children, but also in adults. Multiple clades of EV-D68 have circulated since 2010 in our country, although one (subclade B3) becomes prevalent in 2016 and other (D1) emerges in 2018. In addition, EV-D68 has also been associated with severe neurological cases, indicating the need for better surveillance of this EV type in respiratory specimens at national levels. Further studies are required to understand the mechanisms of this recent emergence and the severe disease presentations involved.
